# Review of the Mechanisms by Which Transcription Factors and Exogenous Substances Regulate ROS Metabolism under Abiotic Stress

**DOI:** 10.3390/antiox11112106

**Published:** 2022-10-25

**Authors:** Peng Liu, Xiaolei Wu, Binbin Gong, Guiyun Lü, Jingrui Li, Hongbo Gao

**Affiliations:** 1 Key Laboratory of North China Water-Saving Irrigation Engineering, Hebei Key Laboratory of Vegetable Germplasm Innovation and Utilization, Collaborative Innovation Center of Vegetable Industry in Hebei, College of Horticulture, Hebei Agricultural University, Baoding 071000, China; 2 Institute of Vegetables Research, Shandong Academy of Agricultural Sciences, Jinan 250100, China

**Keywords:** ROS, abiotic stress, transcription factor, exogenous substances

## Abstract

Reactive oxygen species (ROS) are signaling molecules that regulate many biological processes in plants. However, excess ROS induced by biotic and abiotic stresses can destroy biological macromolecules and cause oxidative damage to plants. As the global environment continues to deteriorate, plants inevitably experience abiotic stress. Therefore, in-depth exploration of ROS metabolism and an improved understanding of its regulatory mechanisms are of great importance for regulating cultivated plant growth and developing cultivars that are resilient to abiotic stresses. This review presents current research on the generation and scavenging of ROS in plants and summarizes recent progress in elucidating transcription factor-mediated regulation of ROS metabolism. Most importantly, the effects of applying exogenous substances on ROS metabolism and the potential regulatory mechanisms at play under abiotic stress are summarized. Given the important role of ROS in plants and other organisms, our findings provide insights for optimizing cultivation patterns and for improving plant stress tolerance and growth regulation.

## 1. ROS Metabolism under Abiotic Stress

Due to human activities, the Earth’s environment has changed dramatically over the past two hundred years, and the frequency of natural disasters such as drought, waterlogging, salinity and high and low temperatures has increased. Various abiotic stresses, such as cold, heat, drought, flood, salt, excessive light, nutrient deficiency, etc., lead to the overproduction of reactive oxygen species (ROS) in plants which cause damage to proteins, lipids and DNA. Plants generate a burst of ROS in response to infection by virulent or even avirulent bacteria, fungi and viruses [1]. ROS may be critical for establishing the hypersensitivity response (HR) of plants following infection and pathogen recognition [2].

ROS are partially reduced or activated derivatives of oxygen (singlet oxygen (^1^O_2_), superoxide anion ($O_{2}^{-}$), hydrogen peroxide (H_2_O_2_), hydroxyl radical (OH·), alkoxy radicals (RO^·^), etc.) that play an important role in plant growth and development. ROS act as a signal to regulate a series of plant biological activities, such as promoting seed germination, inhibiting Arabidopsis thaliana hypocotyl elongation, tissue root structure and flowering regulation [3,4,5,6,7,8,9]. However, excess ROS caused by biotic and abiotic stresses leads to the destruction of macromolecules in plants [10,11]. Thus, to maintain ROS homeostasis, plants have an effective and redundant network of ROS generation and scavenging that involves ROS-producing genes, ROS scavenging-related genes and regulators of these genes.

Here, we mainly introduce the mechanisms of ROS generation and scavenging, review the response laws and mechanisms of ROS metabolism that have recently been studied and discuss the mechanism by which exogenous substances regulate ROS metabolism under abiotic stress. This study provides a reference for the investigation of plant adaptive mechanisms and stress physiology.

**Figure 1:**
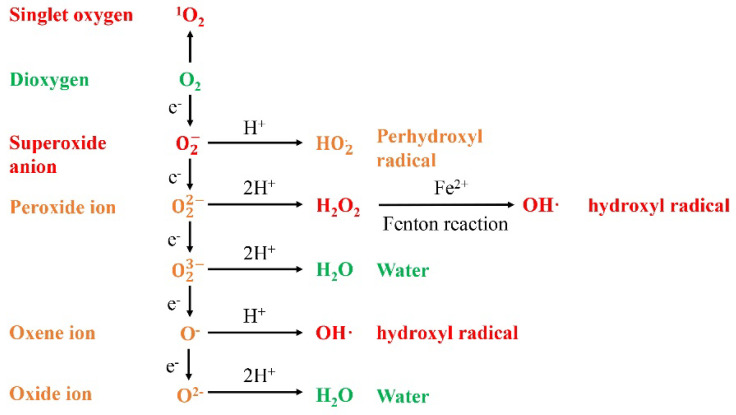
Generation of different ROS via energy transfer or sequential univalent reduction in ground state triplet oxygen. Red font: Singlet oxygen (^1^O_2_), superoxide anion ($O_{2}^{-}$), hydrogen peroxide (H_2_O_2_), hydroxyl radical (OH·) are the main forms of ROS in plants; Orange font: Intermediate products of ROS metabolism; Green font: Dioxygen (O_2_) and Water (H_2_O_2_).

**Figure 2:**
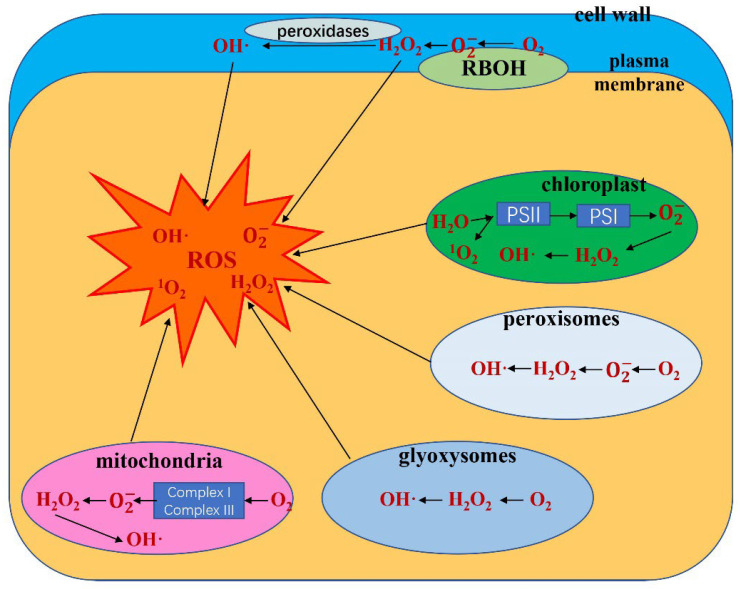
Model of the production and metabolic fate of various ROS (OH·, ^1^O_2_, H_2_O_2_, $O_{2}^{-}$) in different cellular compartments (chloroplasts, mitochondria, peroxisomes, glyoxysomes, plasma membrane and apoplast).

O_2_ preferentially accepts one electron at a time, leading to the production of ROS, which can damage cells in plants. Here, we generalize the process by which oxygen forms different ROS (Figure 1). In general, the production of ROS is accompanied by energy harvest, transmission and consumption, so ROS are mainly produced in mitochondria and chloroplasts and peroxisomes [12]. For example, $O_{2}^{-}$ and ^1^O_2_ are generated in photosystems Ⅰ and Ⅱ (PSI and PSII) of chloroplasts, and $O_{2}^{-}$ is generated in the electron transport chain (ETC) of mitochondria [13]. In addition to mitochondria and chloroplasts, ROS were generated in peroxisomes, glyoxysomes and apoplasts (Figure 2) [14]. To remove excess ROS, the enzymatic and nonenzymatic antioxidant systems in the plant are initiated [15]. In the following section, we summarize the ROS generation and scavenging system in plants to further explicate the regulation of ROS metabolism.

### 1.1. ROS Generation under Abiotic Stress

Plasma membrane RBOH is a key producer of ROS in plants [16]. Therefore, RBOH has been the subject of intensive research [17,18,19]. Ten *RBOH* genes (RbohA to RbohJ) were identified in Arabidopsis thaliana. However, *RBOHs* redundantly work in response to stress. ROS produced by *AtRBOHD* and *AtRBOHF* can be used as signal molecules to improve salt tolerance of Arabidopsis thaliana [20]. RBOH is structurally conserved and consists of two EF-hand motifs, six α-transmembrane helical domains (TMD-I to TMD-VI), an FAD domain and an NADPH domain. RBOH transfers electrons from NADPH to O_2_ to generate $O_{2}^{-}$ via heme in FAD, membranes and TMDs [15]. Chemical inhibitors of RBOH (such as diphenyleneiodonium) have been shown to block or impair ROS generation during biotic or abiotic stress in Arabidopsis thaliana [21]. In contrast, Ca^2+^ directly binds to the EF-hand motif to activate RBOH activity, thereby triggering the generation of ROS [22]. Calcium-dependent protein kinase (CDPK) and Ras-related C3 botulinum toxin substrate (RAC) activate RBOH through the Ca^2+^ pathway [23]. CDPK also directly phosphorylates RBOH [24]. The lipid product phosphatidic acid (PA) of phospholipase Dα1 (PLDα1) can induce *RBOHE,* and *RBOHF* express and activate RBOH and participate in ROS and nitric oxide (NO) production, and NO also regulates the metabolism of ROS as signals in broccoli seedlings [25]. Park et al. found exogenous PA treatment increased ROS levels in Arabidopsis [26]. In addition to RBOH, plant cell wall peroxidases, peroxisomes, and glyoxysomes also catalyze the production of ROS. OH·, H_2_O_2_ and $O_{2}^{-}$ are produced during fatty acid oxidation (by acyl-CoA oxidase) in peroxisomes. H_2_O_2_ and $O_{2}^{-}$ are produced during photorespiration (by glycolate oxidase) in glyoxysomes, respectively [27,28,29]. Cell wall peroxidases catalyze H_2_O_2_ to $O_{2}^{-}$ in cell membranes.

RBOH is a key producer of ROS in plants, and the specific regulation of *RBOH* expression plays an important role in plant resistance to stress [30,31,32]. Kabała et al. reported that *CsRBOHD* and *CsRBOHF1* expression are induced at the early stages of salt stress in cucumber seedlings, and H_2_O_2_ is generated, which functions as a signaling molecule participating in the stress response [33]. This is consistent with the findings of Gémes et al. in tobacco [34]. In cucumber, both salt stress and the maintenance of acquired cold tolerance depend on RBOH [35]. Zhang et al. reported that *CsRbohD* is essential in resistance to cold stress in cucumber. These results suggest that *RBOHD* and *RBOHF* act as ROS signal initiators and activate antioxidant systems during plant resistance to salt and cold stress [36]. Whether *RBOH D* and *E* genes expression is regulated by other abiotic stresses and what the roles of other *RBOH*s are in abiotic stresses may remain to be explored. Another enzyme that responds to abiotic stress is oxalate oxidase. Oxalate oxidase is involved in ROS production in plant root cells during response to drought stress on maize and rice, and heavy metal ions stress on wheat [37,38]. The functions of these oxidases in plant resistance to stress require further study.

Photosynthesis and respiratory metabolism are accompanied by the production of ROS. The capacity for light capture during photosynthesis can exceed use, which can produce $O_{2}^{-}$ and H_2_O_2_ through oxygen photoreduction in PSI and can form singlet oxygen by the interaction of oxygen with triplet-state chlorophyll in PSII [39,40,41]. To reduce the generation of ROS, photosynthetic organisms minimize overexcitation of the photosystems. Nonphotochemical quenching (NPQ) mechanisms that dissipate energy in excess of that used by the photosynthetic electron transport chain are induced to reduce damage to the photosynthetic apparatus caused by the synthesis of ROS [42,43]. In plant mitochondria, the key sources of ROS production are NADH dehydrogenase complexes I and III and the ubiquinone pool [44,45], where $O_{2}^{-}$ radicals are generated from the complexes as a byproduct of energy metabolism by the reduction state of the ubiquinone pool [46,47].

Abiotic stress can result in an oxidative burst and the release of ROS in the photosynthetic and respiratory systems of plants. Abiotic stress triggers a reduction in the respiration rate and inhibition of carbon assimilation, resulting in excess NADPH, which causes excess ROS [48,49]. Plants also respond to adversity stress through a series of complex physiological activities, which regulate the generation of ROS, and many hormones, such as ethylene, jasmonic acid (JA) and salicylic acid (SA), are involved in this process [50]. Hormones are commonly affected by abiotic stress, and these hormones play an important role in plant resistance to stress. Many studies have shown that hormones can regulate the production of ROS in granules and chloroplasts under stressful conditions. It has been reported that abscisic acid (ABA) directly influences photosynthetic oxygen evolution related to the functioning of PSII centers by disrupting the barley chloroplast structure [51]. Meanwhile, ABA increases the content of total carotenoids, xanthophylls and chlorophyll in leaves to ameliorate the impact of excessive excitation energy on PSII. Additionally, ABA downregulates the expression of light-harvesting chlorophyll a/b binding (*LHCB*) genes, *LHCB1-5*, which is beneficial for reducing the absorption of light energy under adverse conditions, thereby reducing the excessive synthesis of ROS in Arabidopsis [52]. Auxin, cytokinin, JA and ethylene may also play important roles in the stability of PSI and PSII, and thus in the improvement of photosynthesis and ROS balance in plants exposed to abiotic stress. During this process, the regulation of *LHCB* gene expression by these hormones is the key activity. Studies have shown that *LHCB* gene expression is regulated by these hormones [50]. However, it is not clear how hormones regulate the expression of these genes. Plant mitochondria may control ROS generation by means of energy-dissipating systems. Therefore, scholars have speculated that mitochondria may play a central role in cell adaptation to abiotic stresses [53]. However, due to a lack of suitable research methods and the specific function of mitochondria, which is energy factory of life and inevitably produces ROS, the mechanism of ROS metabolism in mitochondria under adverse conditions is still unclear.

### 1.2. ROS Scavenging under Abiotic Stress

**Figure 3:**
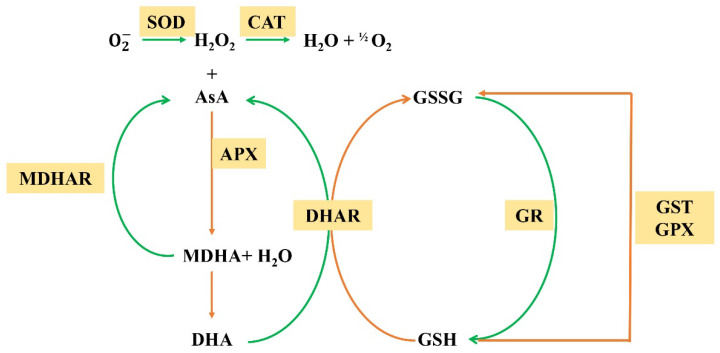
Model of the ASA–GSH cycle. The orange line indicates oxidation reaction and the green line indicates reduction reaction.

The enzymatic ROS scavengers that have been reported thus far include superoxide dismutases (SODs), catalase (CAT), glutathione peroxidase (GPX), ascorbate peroxidase (APX), monodehydroascorbate reductase (MDHAR), glutathione-S-transferase (GST), dehydroascorbate reductase (DHAR) and glutathione reductase (GR). SODs act as the first line of defense against ROS, dismutating $O_{2}^{-}$ to H_2_O_2_. H_2_O_2_ is then detoxified by APX, GPX and CAT [53]. These enzymatic reactions require the participation of ascorbic acid (AsA), dehydroascorbate (MDA), glutathione (GSH), etc. AsA and GSH also regenerate through the ASA–GSH cycle (Figure 3). In addition to enzymatic ROS scavengers, nonenzymatic antioxidants include the major cellular redox buffers AsA and GSH, as well as tocopherol, flavonoids, alkaloids, vitamin E, mannitol, proline and carotenoids [54].

To remove excess ROS in plants under different types of adverse conditions, nonenzymatic and enzymatic antioxidant systems are activated. For example, CAT is required for removing ROS under drought conditions [55], and both SOD and POD play important roles in plant responses to various stresses. Scholars have found that SOD, POD, flavonoids, polyphenols and alkaloids respond to salt stress to scavenge ROS. However, there is controversy about the role of CAT under salt stress [56]. This may be due to the presence of other ROS scavenging systems in plants. Adverse stress induces an increase in the synthesis of plant secondary metabolites, such as flavonoids, polyphenols and alkaloids, which have high medicinal value [57,58,59]. Therefore, many studies have been published on the topic of exposing plants with desirable secondary metabolites to moderate stress in order to increase the quality of black tea and tomato [60,61].

## 2. The Function of Transcription Factors in ROS Metabolism under Abiotic Stress

ROS homeostasis in plants is regulated by a complex and redundant network, and transcription factors (TFs) play an important role in this network. TFs are proteins that can bind to target gene promoters and that regulate the expression of target genes. There are many transcription factors in plants, such as ethylene response factor (AP2/ERF), WRKY, NAC and MYB, that regulate the metabolism of ROS. We found that AP2/ERF, WRKY and NAC mainly regulate the enzymatic antioxidant system, which resists oxidative stress caused by adversity. In addition, bHLH, MYB, bZIP and Dof regulate nonenzymatic antioxidant systems, such as tocopherol, flavonoid, proline and carotenoid metabolism. Undoubtedly, these transcription factor families can regulate both enzymatic and nonenzymatic antioxidant systems. The generation of ROS is also regulated by transcription factors, such as tobacco bHLH123 and Arabidopsis ERF74, which activate *NtRbohE* and *RbohD*, respectively (Table 1) [62,63].

With the popularization of transgenic technology, the functions of many transcription factor genes in plants under abiotic stress have been identified. Many studies have shown that overexpression of *AtWRKY30*, *BdWRKY36*, *SbWRKY30*, *SpWRKY1*, *ZmWRKY40* and *ZmWRKY106* increased the activities of POD, CAT, SOD and some stress related genes (*RD29A*, *HSP90* (*heat shot protein 90*), *DREB2A*, *DREB2B*, *CuZnSOD*, *NCED1* (*Nine cis epoxycarotenoid dioxygenase 1*), *NCED3*, *NCED6*, *LEA5*, *NtP5CS*, and decreased the content of ROS under drought stress [64,65,66,67,68,69]. This indicates that WRKY TFs regulate plant drought resistance by improving the capacity of scavenging ROS. Scholars found that tomato JUNGBRUNNEN1 and SlNAC2 promote *SlDREB1*, *SlDREB2*, *SlDELLA* and glutathione biosynthesis genes to reduce drought-induced accumulation of ROS in tomato, respectively [70,71]. In addition, Niu et al. found that the expression levels of multiple genes regulating ROS production and scavenging were regulated by BnaNAC55 in *Brassica napus* L. [72]. That suggested that NAC TFs regulates plant drought resistance by regulating ROS production and scavenging. Relevant scholars found that NtERF172 and JERF3 induced *NtCAT* and *SOD* expression to decrease drought tolerance through the regulation of CAT-mediated H_2_O_2_ homeostasis in tobacco [73,74]. Much research has shown that GbMYB5 and TaMyb1D regulated the expression of genes encoding antioxidant enzymes and activity of antioxidant enzymes under drought stress in cotton and tobacco, respectively [75,76]. Furthermore, PbrMYB21 of Pyrus betulaefolia could regulate polyamine accumulation, which is also a mechanism by which plants eliminate reactive oxygen species induced by drought stress [77]. This indicated that MYB TFs can activate both enzymatic and nonenzymatic antioxidant systems. Peanut AhbHLH112 and apple *MdbHLH130* act as a positive regulator of drought stress responses through ROS-scavenging in tobacco [73,78]. From these studies, it can be seen that transcription factors can regulate the homeostasis of ROS under drought stress through hormone metabolism and enzymatic antioxidant systems. In addition to WRKY, NAC, AP2/ERF, MYB and bHLH TFs, there are many TFs involved in the regulation of ROS metabolism under drought stress. For instance, TaBZR2 directly interacts with the gene promoter to activate the expression of *TaGST1* to scavenge drought-induced superoxide anions in tobacco [79]. However, the target genes of many transcription factors in the clearing of drought-induced oxidative stress are still unclear.

The lack of oxygen in plants caused by prolonged flooding is the main cause of plant waterlogging. Meng et al. found that 34 WRKY genes were regulated by waterlogging in apple [80]. However, the function of these differentially expressed genes on flood tolerances remains unknown. Liu et al. found that WRKY33 enhanced the expression of hypoxia-responsive genes and alleviated oxidative stress in Arabidopsis [81]. RAP2.12, an ERF TF, induced HRU1, which modulates ROS production in Arabidopsis [82]. Park et al. found that AtERF71/HRE2-overexpressing transgenic Arabidopsis showed tolerance to flooding stress, exhibiting lower levels of ROS [83]. Rice OsEBP89 and Arabidopsis AtERF71 play an important role in alleviating oxidative stress caused by flooding [83,84]. However, the reason for the decrease in ROS is still unknown. At present, there are few published studies on the responses of transcription factors to hypoxia and waterlogging, and much research is still needed.

Salt stress can cause poor plant growth and development. Overexpression of *AhWRKY75*, *FtWRKY46*, *HbWRKY82*, *MfWRKY70* and *PcWRKY33* could improve the salt tolerance of peanut and Arabidopsis [85,86,87,88,89]. The activation of antioxidant enzymes, such as SOD, POD and CAT, is the main reason for the improvement in salt tolerance of WRKY TFs plants. In addition, ROS-related genes (*RbohD*, *CSD1*, *CSD2*, *FSD3*) and hormone signaling genes (*EIN3*, *ABF3*, *ABF4*) are induced in *HbWRKY82* expression plants, and ROS signals and ABA signals may activate antioxidant enzymes. *CaNAC46, GmNAC06, GmNAC065, MbNAC25* and *RtNAC100* play an important role in eliminating ROS induced by salt stress [90,91,92,93,94]. Overexpression of *CaNAC46, GmNAC06, GmNAC065, MbNAC25* and *RtNAC10* induced the antioxidant enzymes genes express, activated antioxidant enzymes and promoted proline accumulation in Arabidopsis, soybean and recretohalophyte Reaumuria trigyna. There have been reports that overexpression of *LcERF056*, *GhERF13.12*, *ZmEREB20* and *SlERF84* are helpful to alleviate oxidative damage induced by salt stress [95,96,97,98]. LcERF056 could bind to cis-element GCC box or DRE of ROS-related genes in Lotus corniculatus [98]. Overexpression of *GhERF13.12* and *ZmEREB20* induced proline biosynthesis and ROS scavenging genes express in upland cotton and Arabidopsis. *SlERF84* gives transgenic tomato a better ROS-scavenging capability. This indicates that ERF TFs can activate the ROS scavenging system. Genetic evidence suggested that *MYB49*, *SlMYB102* and *TaMYB86B* can enhance the ability of tomato and tobacco to scavenge ROS under salt stress [99,100,101]. NtbHLH123 directly regulates *RBOHE* expression and acts as a molecular switch to control an Rboh-dependent mechanism in response to salt stress in tobacco [62]. Peanut AhbHLH112 directly and specifically binds to and activates the promoter of the *POD* gene. *BvBHLH93* induced the expression of antioxidant genes *SOD* and *POD* and repressed the expression of *RbohD* and *RbohF* in Arabidopsis [102]. There are reports that indicated that bHLH TFs can regulate the production and clearance of ROS under salt stress. PeSTZ1 confers salt stress tolerance by regulating the expression of *PeZAT12* and *PeAPX2* in poplar [103]. Many TFs respond to salt stress and regulate ROS metabolism, and many studies have revealed that the expression of these transcription factors can cause changes in the ROS level [90,99]. However, the mechanism by which TFs regulate the metabolism of ROS under salt stress remains unclear.

Appropriate temperature is a necessary condition for plant growth. Low temperature will cause damage to plants and can even cause production failure in severe cases. Fei et al. found that *KoWRKY40* transgenic Arabidopsis exhibited higher proline content, SOD, POD and CAT activities, and lower H_2_O_2_ content than wild-type Arabidopsis under cold stress conditions [104]. *CaNAC064*, *MbNAC25* improved SOD, POD and CAT activities, and scavenging capability of ROS in peppers and Arabidopsis [94,105]. He et al. reported that PeSTZ1 enhances freezing tolerance through modulation of ROS scavenging by directly regulating *PeAPX2* in poplar [103]. Additionally, MYB, ERF and bHLH TFs can directly regulate the expression of ascorbic acid, flavonoid, phenol and anthocyanin synthesis genes to resist low temperature-induced oxidative stress [106,107,108,109]. During this process, the expression of these transcription factors is regulated by hormonal signals such as ethylene. This has important implications for our full comprehension of plant resistance to cold stress.

As global temperatures rise, high or extreme heat is becoming increasingly common. *ZmWRKY106*, *ERF74* and *MYB44* activated antioxidant enzymes, and play an important role in scavenging ROS under heat stress on maize, Arabidopsis and Xanthoceras sorbifolium [64,69,110]. Singh et al. reported that under heat stress, *EcDREB2A* overexpression also resulted in increased antioxidant enzymes activity with decreased ROS content in tobacco [111]. Heat shock transcription factors (HSFs) regulate the expression levels of heat shock proteins and play an important role in plant high temperature stress. LlHsfA4 upregulated *APX2* expression to resist heat stress in lilies [112]. However, ZmHsf08 negatively regulates abiotic stress responses of maize [113]. ZmWRKY106, BZR1, OsSPL7 and other transcription factors play an important role in ROS balance under heat stress [69,114,115]. This showed that TFs could alleviate the oxidative damage caused by high temperatures by activating antioxidant enzymes. However, most of the recent research on this topic only covers the function of these transcription factors; the specific functional mechanism is still unclear.

Multiple abiotic stresses occur simultaneously in the natural environment. Many transcription factors can help plants cope with multiple abiotic stresses that occur simultaneously. *HbWRKY82*, *CaNAC46*, *MYB49*, *AhbHLH112*, *MfbHLH38* and *MfPIF1* could alleviate oxidative damage caused by drought and salt stresses [78,88,92,100,116,117]. *ZmWRKY106* and *MYB44* also responded to drought and heat stresses [69,110]. *ERF74*, *75* also help plants cope with drought, heat, excessive light and aluminum stresses [63].

The complex transcriptional regulatory network of ROS metabolism composed of these transcription factors plays an important role in the resistance to various abiotic stresses. However, the mechanism by which these transcription factors regulate ROS metabolism needs further exploration, which will provide new ideas for solving problems such as the crop yield reduction caused by abiotic stress.

## 3. The Mechanism by Which Exogenous Substances Regulate ROS Metabolism

Although plants have an efficient ROS regulation system, ROS are still inevitably produced in large amounts during an oxidative burst when plants are under stress conditions, and plants are inevitably damaged by ROS. Therefore, exploring the mechanism of ROS metabolism regulation via exogenous substances is of great significance for improving cultivated plant resistance to stress.

### 3.1. Plant Growth Regulators

#### 3.1.1. Epibrassinolide (EBR)

Brassinolide (BRs) is an important phytosterol hormone, which regulates plant growth and development and improves plant resistance to abiotic stresses. In view of the low content of plant-synthesized brassinolide, cheap and efficient synthetic EBR is applied to agricultural production. Fan et al. found that application of EBR can regulate the xylem development of masson pine and accelerate its timber formation [138]. Zhang et al. found that application of EBR is good for the leaf size and expansion of tobacco [139]. Studies have shown that exogenous BR improves the activities of SOD, CAT, POD and APX; promotes the accumulation of AsA and GSH; and increases Fv/Fm, Φ (PSII) and qP [140,141,142]. Exogenous EBR regulates endogenous hormones by activating BR biosynthetic genes at the transcript level, which increases antioxidant enzyme capacity levels and reduces the overproduction of ROS [143]. A BR receptor, BRASSINOSTEROID-INSENSITIVE 1 (BRI1), negatively regulates antioxidant capacity [144]. BRASSINAZOLE RESISTANT 1 (BZR1), the critical regulator of the BR response, binds to the promoters of *FERONIA2* (*FER2*) and *FER3* and induces their expression. BZR1 regulates ROS scavenging through *RBOH1*-dependent ROS signaling, which is at least partially mediated by FER2 and FER3 [114]. Although it has long been reported that exogenous EBR can improve plant antioxidant capacity, a comprehensive analysis of the regulation of ROS metabolism by exogenous EBR still remains a challenge.

#### 3.1.2. GR24

Strigolactone (SL) is a new type of plant hormone that plays an important role in the regulation of lateral growth and ROS metabolism in plants. GR24 is used in agricultural production as an artificial strigolactone analog. Several studies shown that GR24 facilitates light harvest and accumulation of anthocyanins in grapevine berries and tomato [145,146]. GR24 strengthens the enzyme activities of SOD, POD and CAT; promotes the accumulation of proline, GSH, AsA and GABA; and enhances the electron transport rate in PSII and PSI, the nonphotochemical quenching, the oxidized plastoquinone pool size and the ratio of the quantum yield of cyclic electron flow to Y (II) [147,148,149,150]. Interestingly, the AsA content in plants in turn affects endogenous SL metabolism in rice [151]. SL is believed to function in conjunction with ABA and JA [152,153]. NO signaling plays important roles in SL-regulated ROS metabolism [147]. However, the detailed mechanism by which SL regulates ROS metabolism is not fully clear. Furthermore, the appropriate SL dose application for different crops is also not clear.

#### 3.1.3. Abscisic Acid (ABA)

Abscisic acid (ABA), as a hormone, has been intensively studied in order to elucidate its role in plant growth and material metabolism regulation and defense against abiotic stresses. Exogenous application of ABA reduces cold-induced oxidative stress by enhancing the activities of both enzymatic and nonenzymatic antioxidants in maize [154]. The cross-talk of both ABA and NO is believed to increase oxidative stress tolerance in plants, and NO may act downstream of ABA [155]. Furthermore, ABA triggers NO production and enhances counteracting oxidative stress [156]. However, how ABA regulates NO production to relieve oxidative stress still needs to be clarified. In contrast, in Arabidopsis, exogenous ABA inhibits AsA synthesis to promote the accumulation of ROS [157]. Therefore, the effect of exogenous ABA on the metabolism of ROS needs to be further explored.

#### 3.1.4. Salicylic Acid (SA)

Salicylic acid (SA), as a hormone, has been intensively studied in order to elucidate its role in plant growth and material metabolism regulation and defense against biotic and abiotic stresses. Schussler et al. proposed that abscisic acid (ABA) may stimulate sucrose transport into filling seeds of legumes, potentially regulating seed growth rate [158]. Wang et al. reported that exogenous ABA application promotes anthocyanin and sugar accumulation in grape berry [159]. Furthermore, many studies have shown that SA induces the generation of ROS by inhibiting mitochondrial complex III enzymatic activity and by activating RBOH [160,161]. In addition, SA blocks electron flow from the substrate dehydrogenases to the ubiquinone pool and triggers H_2_O_2_ generation [162]. Meanwhile, SA directly binds to CAT and APX, inhibiting their activities in tobacco and mammalian [163]. Subsequently, the ROS generated by SA activation acts as a signal, enhancing *StSABP2*, *StSOD* and *StAPX* expression and SOD, POD and CAT activities and upregulating the ASA-GSH cycle [39,164,165,166,167,168]. Therefore, the novel action of SA in ROS metabolism will likely continue to be unveiled.

#### 3.1.5. Ethephon

Ethylene (ETH) is the only known gaseous plant hormone, so exogenous liquid ethephon, rather than gaseous ETH, has been used for plant growth regulation. Exogenous ethylene application could promote female flower differentiation of horticultural crops. Exogenous ethylene promotes mango fruit peel color transformation by regulating the degradation of chlorophyll and synthesis of anthocyanin and fructan accumulation in chicory [169,170]. Some scholars have found that exogenous application of ethephon can increase the content of H_2_O_2_ and can increase the activity of antioxidant enzymes and the content of antioxidant substances [74,171,172,173,174,175]. Jiang et al. found that ETH signaling upregulates RBOH expression, and that ROS accumulate in *RBOH* mutants. These findings suggest that ETH signaling has the potential to enhance antioxidant system activity by activating ROS signaling. Too much ETH, however, can cause oxidative stress [176]. Ethylene’s ability to regulate ROS metabolism has been widely studied, but the effect of ETH doses on ROS metabolism is still unclear. Moreover, the precise molecular mechanisms by which ETH tunes the ROS scavenging and ROS production machinery to maintain proper ROS levels remain unclear.

### 3.2. Inorganic Substances

#### 3.2.1. Ca^2+^

As an essential medium element of plants, calcium participates in many life processes. Hou et al. found that exogenous Ca^2+^ application had a significant effect on *Brassica napus* height, root length, biomass accumulation and root structure formation, especially on the growth and development of coarse roots [177]. Ca^2+^ is the universal secondary messenger in plant stress signaling. Many studies have shown that exogenous Ca^2+^ is beneficial for plants in scavenging ROS [178,179,180]. In general, exogenous calcium stimulates the production of ROS, which acts as a signal to activate the ROS scavenging system in plants to avoid oxidative damage [36]. Calcium ions enhance RBOH activity and promote the production of plasma membrane ROS, which act as a signal to activate antioxidant enzymes (i.e., POD, CAT and SOD) and the antioxidant system of the AsA-GSH cycle in potato tuber [181]. Moreover, the Ca^2+^/calmodulin system activates glutamate decarboxylase (GAD) in the cytosol, and concomitantly, gamma-aminobutyric acid (GABA) levels increase of soybean [182]. GABA, as a signal, can activate the ROS scavenging system in plants. Meanwhile, exogenous calcium alleviates PSII photoinhibition caused by ROS mainly by promoting carbon fixation, CEF, xanthophyll cycles, PQ pools, and ATPase activity, which affects the oxidative bursts that produce large amounts of ROS, as mentioned in Section 1.1 [183]. However, the mechanism by which calcium regulates GABA and the photosynthetic system still needs to be elucidated. In addition, calcium also directly regulates the ROS scavenging system. A Ca^2+^-sensor, RESISTANCE OF RICE TO DISEASES1 (ROD1), directly scavenges ROS via catalase activation in Arabidopsis [184].

#### 3.2.2. Sodium Nitroprusside (SNP)

Many studies have shown that exogenous nitric oxide (NO) provides protection against oxidative membrane damage for photosynthetic pigments, AsA-GSH and proline [185,186,187,188,189,190]. Notably, SNP acts as a donor of exogenous NO. NO is not only an ROS scavenger itself but also regulates the metabolism of ROS by functioning as a signal [191,192]. As a signal, NO combines with GSH to form *S*-nitrosoglutathione (GSNO) [193]. GSNO is transported in the phloem and activates the antioxidant system in the plant [194]. Transcriptome analysis has found that exogenous NO downregulates RBOH expression and upregulates *CAT*, *POD* and antioxidant synthesis gene expression in alfalfa seedlings [195]. After overexpression of the neuronal NO synthase gene *nNOS*, the expression levels of zinc finger protein transcription factors and C-repeat binding transcription factor (CBF) transcription factors increased, which indicated that zinc finger protein transcription factors and CBF transcription factors may be involved in NO regulation of the expression of antioxidant-related genes. Meanwhile, two ABA receptor genes, *AtPYL4* and *AtPYL5,* are also involved in NO-induced ROS scavenging in Arabidopsis [196]. However, the mechanism by which exogenous NO can improve the antioxidant capacity of plants still requires further research.

#### 3.2.3. Other Inorganic Substances

Jia et al. reported that exogenous phosphorus reduced lipid peroxidation effects on SOD, CAT and POD activities of perennial ryegrass [197]. On the other hand, phosphorus also affects the plant photosynthesis system and thus affects the generation of ROS. H_2_S is another inorganic substance of interest, as it can enhance plant tolerance to salt and heavy metal stresses by regulating Na^+^/K^+^ homeostasis and the uptake and transport of metal ions. H_2_S also promotes the H_2_S-Cys cycle balance under abiotic stress and enhances the roles of the cycle in the regulation of the antioxidant system in the alternative respiratory pathway, and in heavy metal chelator synthesis [198].

### 3.3. Organic Substances

#### 3.3.1. Gamma-Aminobutyric Acid (GABA)

As a signaling molecule, GABA can regulate many physiological processes, including growth, development and stress responses. Xie et al. found that GABA negatively regulates adventitious root development in poplar and respiratory rate titratable acidity of apple but improves morphological growth of maize [199,200,201]. Many studies have shown that exogenous GABA elevates plant stress tolerance by improving photosynthesis, inhibiting ROS generation, and activating antioxidant enzymes [202,203,204,205,206]. Exogenous GABA application induces an increase in endogenous GABA. The potential mechanisms by which exogenous GABA alleviates oxidative injury may be related to the enhancement of plant antioxidant systems, which results in reductions in malondialdehyde (MDA) and ROS levels, and to proline accumulation-mediated osmoregulation. In contrast, Bouche et al. reported that in GABA-deficient mutant Arabidopsis with the *succinic semialdehyde dehydrogenase* (*ssadh*) phenotype, the level of ROS was significantly increased, and the mutants behaved abnormally and were more sensitive to stress [207]. Wang et al. proposed a potential mechanism whereby exogenous GABA mitigates oxidative damage caused by hypoxia in melon plants by accelerating polyamine biosynthesis and transformation to prevent polyamine degradation [205]. It has also been reported that exogenous GABA plays an antioxidant role by promoting *glutathione transferase* (*GST*) gene expression and enhancing GSH peroxidase activity to catalyze the binding reaction of GSH with various electrophilic exogenous chemicals. However, how GABA activates the ROS scavenging system remains unclear.

#### 3.3.2. Polyamines

Polyamines, putrescine (Put), spermidine (Spd) and spermine (Spm), are widely present in plants and are closely related to plant growth and development. Qu et al. found that exogenous Put significantly ameliorated the losses of Chl and improved photochemical capability and prevented membrane impairment of *Nephrolepis cordifolia* [208]. Tavallali et al. reported exogenous Spd significantly increased levels of phenolic and flavonoid compounds in pot marigold [209]. Polyamines can not only scavenge ROS directly through disproportionation reactions [210] but can also increase the content of antioxidant substances by increasing the activity of antioxidant enzymes [211]. Moreover, exogenous application of polyamines can maintain the activity of PSII of helianthus tuberosus and can reduce the production of ROS in PSI at the end of the chloroplast electron transport chain [212]. Moreover, polyamines inhibit RBOH activity and reduce the accumulation of hydrogen peroxide of cucumber cultivars [213]. However, the mechanism by which polyamines act as signals to activate the antioxidant system and regulate the expression of RBOH still needs further exploration.

#### 3.3.3. Melatonin

Melatonin is a small molecule indoleamine substance, which has important functions in animals and plants. Xiao et al. found that 20 μM melatonin treatment optimally promotes cotton seed germination [214]. Melatonin increases the activities of various antioxidant enzymes and the levels of antioxidants such as AsA, GSH and phenolic compounds, thereby reducing ROS accumulation and alleviating oxidative damage induced by drought, chilling and aluminum stress [215,216,217]. However, the mechanism by which melatonin, as a signaling substance, regulates the metabolism of ROS under abiotic stress still needs to be further explored.

#### 3.3.4. Sugars

Many studies have shown that sucrose, trehalose and chitosan can alleviate oxidative damage caused by abiotic stress [218,219,220,221]. Van den Ende et al. reported that sucrose and sucrose oligosaccharides are involved in stabilizing membrane-associated peroxidases and NADPH oxidases in Arabidopsis [219]. Trehalose plays an important role in resistance to adversity and abiotic stress, and these reports indicate that trehalose can enhance antioxidant systems, activate photosynthesis and protect cell structure [222,223]. Wang et al. reported that exogenous chitosan can improve photosynthetic capacity and antioxidant enzyme activity of banana plants and can reduce ROS induced by chilling injury [224]. Sugars are easy to obtain, are inexpensive and can be widely used in agricultural production. Therefore, it is important to analyze the mechanism by which sugars regulate ROS metabolism under stress. Furthermore, Tan et al. found exogenous sucrose could effectively promote vitamin C, sucrose and fructose contents of pea sprouts [225].

#### 3.3.5. Other Organic Substances

Exogenous proline, L-carnitine, p-coumaric acid and GSH can alleviate oxidative damage caused by abiotic stress on salvia hispanica, Arabidopsis and barley seedlings [226,227,228,229]. However, the mechanism by which these exogenous substances regulate the metabolism of ROS under adverse conditions still needs to be further explored.

## 4. Conclusions and Perspectives

**Figure 4:**
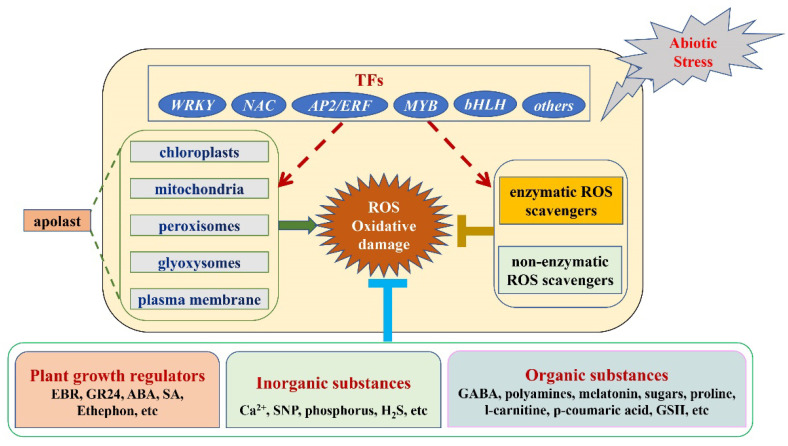
The model of transcription factors and exogenous substances regulate ROS metabolism under abiotic stress.

Because ROS play a dual role in plants, both as toxic byproducts and as signaling molecules, the ROS synthesis and ROS scavenging machineries are tightly regulated to achieve appropriate levels of ROS at different developmental stages and in different growing environments. However, with the deterioration of the natural environment, abiotic and biotic stresses such as drought, waterlogging, salinity and alkalinity disrupt the ROS balance in plants, which causes oxidative stress to the plant. Through transgenic technology, we can regulate the expression of TFs, reduce the oxidative damage caused by abiotic stress and improve the ability of plants to resist abiotic stress. On the other hand, the use of exogenous substances to regulate the generation and scavenging of ROS in plants has been widely studied with the aim of alleviating oxidative damage caused by biotic and abiotic stresses in cultivated plants (Figure 4). However, many questions remain unanswered, among which are the following:

(1) Are there previously undiscovered forms of ROS and ROS generation and scavenging systems in plants?

(2) Although many transcription factors have been found to be involved in the regulation of ROS metabolism resistance to abiotic stress, specifically how they regulate ROS metabolism to resist abiotic stress is still unclear, and much work is still needed in this area.

(3) Although most of the aforementioned studies have shown that many exogenous substances can regulate the metabolism of ROS in plants in response to abiotic factors, there are some differences in the findings, which may be due to differences in the plant species and the amount of exogenously applied substances used in the studies. These divergent results require further investigation. The economic and environmental sustainability of applying these exogenous substances also needs to be considered. Most recent studies in this area have been on the effects of a single exogenous substance on the regulation of ROS. There are few reports on the effects of two or more exogenous additives on plant resistance to oxidative stress and ROS production; thus, additional research is needed. Furthermore, it is of great value to elucidate more types of exogenous substances and comprehensively evaluate their effects on the generation and scavenging of ROS in plants.

Such studies will further deepen our understanding of the role of ROS metabolism under abiotic stress in plants and may provide insights for developing new cultivation models.

## Figures and Tables

**Table 1:** Transcription factors that participate in the metabolism of ROS.

Transcription Factor Family	Genes Name	Description
WRKY	*AhWRKY75* [85]	Peanut *AhWRKY75* gene conferred salt tolerance in transgenic peanut lines by improving the efficiency of the ROS scavenging system and photosynthesis.
	*EjWRKY17* [118]	Overexpression of Eriobotrya japonica *EjWRKY17* led to enhanced drought tolerance in transgenic Arabidopsis, which was lower levels of ROS.
	*FtWRKY46* [89]	Overexpression of Tartary buckwheat *FtWRKY46* enhanced the stress tolerance of transgenic Tartary buckwheat by modulating ROS clearance and stress-related gene expression.
	*HbWRKY82* [88]	Hevea brasiliensis *HbWRKY82* regulated the transcriptional expression of ROS-related genes (*RbohD, CSD1, CSD2, FSD3*) against salt and drought stress in Hevea brasiliensis.
	*KoWRKY40* [104]	Mangrove plant K. obovate *KoWRKY40* transgenic Arabidopsis exhibited higher proline content, SOD, POD and CAT activities, and lower H_2_O_2_ content under cold stress conditions.
	*MfWRKY70* [87]	Myrothamnus flabellifolia *MfWRKY70* could significantly increase tolerance to drought, osmotic and salinity stresses by enhancing the antioxidant enzyme system and maintaining ROS homeostasis in Myrothamnus flabellifolia.
	*PcWRKY33* [86]	Polygonum cuspidatum *PcWRKY33* negatively regulates the salt tolerance by increasing the level of cellular ROS in Arabidopsis thaliana.
	*WRKY33* [119]	Arabidopsis thaliana *WRKY33* can bind to and activate *RAP2.2* and activate towards *RAP2.2* to increase hypoxia tolerance of Arabidopsis thaliana.
	*ZmWRKY40* [68]	Overexpression of maize *ZmWRKY40* improved drought tolerance in transgenic Arabidopsis by enhancing the activities of POD and CAT under drought stress.
	*ZmWRKY79* [120]	Maize *ZmWRKY79* boost ROS scavenging to result in less H_2_O_2_ and MDA accumulation and increased antioxidant enzyme activities under drought stress in Arabidopsis.
	*ZmWRKY106* [69]	Overexpression of maize *ZmWRKY106* improved the tolerance to drought and heat in transgenic Arabidopsis by reducing ROS content in transgenic lines by enhancing the activities of SOD, POD and CAT under drought stress.
NAC	*CaNAC46* [92]	Overexpression of Capsicum annuum *CaNAC46* improved the tolerance of transgenic Arabidopsis thaliana plants to drought and salt stresses by promoting the expression of *SOD* and *POD*.
	*CaNAC064* [105]	The Capsicum annuum *CaNAC064*-overexpressing Arabidopsis plants exhibited lower MDA content, chilling injury index under cold stress.
	*GmNAC06* [90]	Soybean GmNAC06 could cause the accumulation of proline and glycine betaine to alleviate or avoid the negative effects of ROS in soybean.
	*GmNAC065* [93]	Soybean *GmNAC065* expression shows a phenotype associated with enhanced oxidative performance and higher carotenoid contents under salt stress in Arabidopsis.
	*GmNAC085* [121]	Soybean *GmNAC085* mediated drought resistance in transgenic Arabidopsis plants, with higher activities of antioxidant enzymes responsible for scavenging hydrogen peroxide or superoxide radicals.
	*MbNAC25* [94]	Overexpressing *Malus baccata* (L.) Borkh *MbNAC25* Arabidopsis plants showed enhanced tolerance against cold and drought salinity by increasing proline content, the activities of antioxidant enzymes SOD, POD and CAT.
	*ONAC066* [122]	Overexpression of rice *ONAC066* in transgenic rice improved drought and oxidative stress tolerance, accompanied with increased contents of proline, decreased accumulation of ROS in rice.
	*RtNAC100* [91]	R. trigyna *RtNAC100* overexpression aggravated salt-induced PCD in transgenic R. trigyna lines by promoting ROS.
AP2/ERF	*ERF6* [123]	Arabidopsis ERF6 functions as a transcriptional activator and suppressor of genes in response to drought stress and decreased ROS content in Arabidopsis.
	*ERF96* [124]	Arabidopsis *ERF96*-overexpressing Arabidopsis lines exhibited the significant increases in CAT and GPX activities as well as the glutathione (GSH) content, while having a decrease in ROS accumulation compared to WT.
	*ERF74* [63]	Arabidopsis *ERF74*-overexpressing Arabidopsis lines showed enhanced tolerance to drought, high light, heat and aluminum stresses, and induction of stress marker genes and ROS-scavenging enzyme genes is dependent on the *ERF74-RbohD*-ROS signal pathway.
	*LcERF056* [98]	Lotus corniculatus *LcERF056* plays important roles in salt tolerance in Lotus corniculatus by modulating ROS-related genes
	*GhERF13.12* [96]	*GhERF13.12* from Gossypium hirsutum transgenic Arabidopsis showed enhanced salt stress tolerance and enhanced expression of genes participating in proline biosynthesis, and ROS scavenging.
	*ZmEREB20* [95]	Maize *ZmEREB20* positively regulated salt tolerance through the molecular mechanism associated with ROS scavenging in maize.
	*SlERF84* [97]	Overexpression of tomato *SlERF84* in Arabidopsis endows transgenic plants enhanced tolerance to drought and salt stress.
MYB	*CmMYB012* [125]	*CmMYB012* from Chrysanthemum morifolium was also found to inhibit anthocyanin biosynthesis by suppressing the expression of *CmCHS, CmDFR, CmANS* and *CmUFGT* against heat stress on Chrysanthemum morifolium.
	*MdMYB23* [126]	Transgenic apple calli and Arabidopsis with overexpression of *MdMYB23* from apple exhibited increased cold tolerance through active *MdANR* to promote proanthocyanidin accumulation and ROS scavenging.
	*MYB44* [110]	Suppression of *XsMYB44* expression via virus-induced gene silencing weakened yellowhorn tolerance to both individual and combined drought and heat stress and increased ROS levels and decreased antioxidant enzyme activities and proline content.
	*MYB49* [100]	Overexpression of *SlMYB49* in tomato significantly enhanced the resistance of tomato to salt and drought stress and decreased accumulation of ROS.
	*PlMYB108* [127]	Overexpression of *PlMYB108* from Herbaceous Peony in tobacco plants, showed that the flavonoid content, antioxidant enzyme activities, and photosynthesis were markedly elevated to confer drought stress.
	*SlMYB102* [99]	The overexpression of *SlMYB102* in tomato maintained lower ROS generation and increased the activity of ROS scavenging enzymes, the accumulation of antioxidants and proline was higher under salt stress.
	*TaMYB86B* [128]	Wheat *TaMYB86B* influences the salt tolerance of wheat by regulating the ion homeostasis to maintain an appropriate osmotic balance and decrease ROS levels.
bHLH	*AhbHLH112* [78]	The overexpression of *AhbHLH112* from peanut improves the drought tolerance of transgenic Arabidopsis plants both in seedling and adult stages through directly activating the *POD* gene.
	*bHLH123* [62]	Overexpression of *NtbHLH123* from tobacco resulted in greater resistance to salt stress on tobacco through the *NtbHLH123-NtRbohE* signaling pathway.
	*BvbHLH93* [102]	Overexpression of sugar beet B*vBHLH93* in Arabidopsis enhanced the activities of antioxidant enzymes by positively regulating the expression of antioxidant genes SOD and POD to against to salt stress.
	*MdbHLH130* [73]	Overexpression of apple *MdbHLH130* in tobacco led to lower ROS accumulation and upregulation of the expression of some ROS-scavenging under drought stress.
	*MfbHLH38* [116]	Heterologous expression of M. flabellifolia *MfbHLH38* in Arabidopsis improved the tolerance to drought and salinity stresses, decreased proline and ROS accumulation and increased antioxidant enzyme activities
	*MfPIF1* [117]	Overexpression of *MfPIF1* from M. flabellifolia in Arabidopsis thaliana led to enhanced drought and salinity tolerance, which was attributed to higher contents of proline and activities of antioxidant enzymes, as well as lower ROS accumulation in transgenic lines.
	*OsWIH2* [129]	Heterologous expression of *OsWIH2* in rice resulted in significantly higher drought tolerance, probably due to the decreased ROS accumulation under drought stress.
	*PYE, ILR 3* [130]	Arabidopsis *ILR3* and *PYE* confer photoprotection during Fe deficiency to prevent the accumulation of singlet oxygen and repair of the photosynthetic machinery.
Other families	*CaSBP12* [131]	Silencing the *CaSBP12* gene enhanced pepper plant tolerance to salt stress and decreased accumulation of ROS.
	*CsHB5* [132]	Heterologous expression of *CsHB5* in citrus calli upregulated the expression of ROS-related genes and increased the content of H_2_O_2_ to against to senescence.
	*HY5* [133]	Arabidopsis *ELONGATED HYPOCOTYL5* as a major transcription factor required for activation of the detoxification program under high N.
	*MdZAT10* [134]	Heterologous expression of *MdZAT10* in apple calli decreased the expression level of *MdAPX2* and increased sensitivity to drought stress.
	*MdHB7-like* [135]	Heterologous expression of *MdHB7-like* reduced ROS under salt stress.
	*OsMADS57* [136]	Overexpression of rice *OsMADS57* in both Arabidopsis thaliana and rice could improve their salt tolerance by increasing the activities of antioxidative enzymes.
	*ZAT18* [137]	Heterologous expression of *ZAT18* in Arabidopsis improved drought tolerance and exhibited a lower content of ROS and higher antioxidant enzyme activities.
